# Myocardial apoptosis and mesenchymal stem cells with acute exercise

**DOI:** 10.14814/phy2.13297

**Published:** 2017-06-02

**Authors:** Maria F. Arisi, Erica N. Chirico, Roxanne Sebeny, Geetha Muthukumaran, Anbin Mu, Bart C. De Jonghe, Kenneth B. Margulies, Joseph R. Libonati

**Affiliations:** ^1^ School of NursingUniversity of PennsylvaniaPhiladelphiaPennsylvania; ^2^ Cooper Medical SchoolRowan UniversityCamdenNew Jersey; ^3^ Perelman School of MedicineUniversity of PennsylvaniaPhiladelphiaPennsylvania

**Keywords:** Acute exercise, apoptosis, heart, mesenchymal stem cells

## Abstract

Aerobic exercise confers many health benefits. However, numerous reports have shown that acute aerobic exercise can injure the heart. We tested the general hypothesis that acute moderate‐intensity exercise in rodents induces cardiomyocyte damage and stimulates mesenchymal stem cells (MSCs) to increase paracrine‐mediated protective effects on cardiomyocytes. A single session of treadmill running (13 m/min, 0% grade, for 45 min) in untrained C57BL/6 male mice (*n* = 18) increased cleaved poly ADP‐ribose polymerase (PARP), a marker of apoptosis, in the myocardium 24 h postexercise. Microarray analysis of mouse myocardium identified 11 relevant apoptotic genes and several shifts in matrix remodeling transcripts over the postexercise window. Postexercise cardiomyocyte death was recapitulated in neonatal rat cardiomyocytes (NRCMs) by culturing cells in 2% plasma harvested from exercised rats. The increased cell death observed in exercise‐treated NRCMs was attenuated by *β*‐adrenergic blockade, but not antioxidant treatment. MSC survival, proliferation, and chemotaxis showed no significant differences between sedentary and exercise plasma conditions, despite increased IL‐6, TNF‐*α*, IL‐1*β*, and IFN‐*γ* secretions from MSCs treated with exercise plasma. NRCM survival was increased nearly 500% when cocultured with MSCs, but this effect was not altered under exercise plasma culture conditions. Our results suggest acute moderate‐intensity aerobic treadmill running in exercise‐naïve rodents induces temporal cardiomyocyte death due to plasma‐borne factors, namely, catecholaminergic stress. Even though exercise conditions prompt an inflammatory response in MSCs, the exercise milieu does not alter the MSC‐protective phenotype on cardiomyocytes.

## Introduction

Several studies have shown that the heart exhibits cardiomyocyte renewal mechanisms that persist throughout the life span (Bergmann et al. 2009; van Berlo et al. 2014). While cardiomyocyte renewal is a multifactorial process, stem cells are thought to play a regulatory role (van Berlo et al. 2014). In this context, mesenchymal stem cells (MSCs) are multipotent and immune privileged stem cells capable of triggering the production of reparative growth factors, modulating inflammation, and creating an overall favorable microenvironment for endogenous cardiac repair (Aggarwal and Pittenger 2005; Nesselmann et al. 2008; Boyle et al. 2016; Gao et al. 2016). As MSC therapeutic potency is known to be stimulated after deleterious stimuli such as hypoxia and inflammation, their response to physiological stresses such as acute exercise has received limited attention. This is a significant issue because acute aerobic exercise has been shown by unknown mechanisms to induce transient contractile dysfunction and injury in the heart and, hence (Middleton et al. 2008; Scharhag et al. 2008; Shave et al. 2010), may stimulate the reparative effects of MSCs. Moreover, exercise is increasingly being investigated as an adjuvant to exogenous stem cell therapy (Wiskemann and Huber 2008; Wiskemann et al. 2011; Emmons et al. 2016).

Several data have shown that MSCs mobilize from bone marrow in response to exercise (Valero et al. 2012; Keser et al. 2013), and that MSCs released postexercise have increased osteogenic potential and can promote angiogenesis in the myocardium (Kwon et al. 2014; Marędziak et al. 2015). In order for MSCs to exert a therapeutic effect, these cells must acquire adhesive interactions with the vascular endothelium, a process that has also been shown to change with exercise (Niebauer and Cooke 1996; Jee and Jin 2012). Once retained in the myocardium, MSCs improve the survival, bioenergetics, and the function of host tissue (Naji et al. 2017), largely through paracrine signaling (Liang et al. 2014). How these putative functions of MSCs are altered in the postexercise metabolic milieu remains unknown. In this study, we tested the hypothesis that acute aerobic exercise in rodents induces cardiomyocyte damage and stimulates the paracrine‐mediated protective effects of MSCs on cardiomyocytes. Specifically, we established: (1) the temporal myocardial apoptotic molecular changes associated with a single bout of moderate‐intensity treadmill running, (2) the role of catecholamines and free radicals in postexercise cardiomyocyte death, and (3) the protective influences of MSCs on cardiomyocyte survival under in vitro conditions.

## Materials and Methods

### Animal ethics

All experimental protocols were approved by the Institutional Animal Care and Use Committee of University of Pennsylvania and were conducted in accordance with the National Institute of Health Guide for the Care and Use of Laboratory Animals (NIH Publications No. 80–23, revised in 1996).

### Acute exercise and temporal apoptosis in murine myocardium

Six‐ to eight‐week‐old male C57BL/6 mice (*N* = 18, Jackson labs) were housed in temperature‐controlled conditions with a 12‐h light/dark cycle, and were fed water and mouse chow ad libitum throughout the study period. All mice were acclimated at standard cage conditions for at least 72 h before exercise treatment and all animals were maintained with institutional standards and in accord with American Physiologic Society Guidelines. Mice were exercised on a motorized treadmill at 13 m/min, 0% grade, for 45 min or remained sedentary. This exercise intensity is equivalent to 60–70% VO_2_ peak (Fernando et al. 1993; Schefer and Talan 1996). To study temporal patterns of myocardial apoptosis, mice were euthanized at sedentary baseline (*N* = 3) or immediately postexercise (*N* = 3), 8 h postexercise (*N* = 3), 24 h postexercise (*N* = 3), 48 h postexercise (*N* = 3), and 72 h postexercise (*N* = 3).

### Gene microarray and Western blots

After flushing the excised heart with PBS, right and left ventricles were separated, frozen, and pulverized with mortar and pestle, and RNA and protein isolated using the AllPrep DNA/RNA/Protein mini kit (80004; Qiagen, Valencia, CA). RNA was quantitated spectrophotometrically in a Nanodrop instrument. One microgram of mRNA was converted into cDNA using the RT^2^ First Strand Kit (330401, Qiagen). The mouse RT^2^ Profiler Apoptosis PCR array (PAMM‐012Z, Qiagen) was employed to determine the relative expression levels of genes in the apoptotic pathway, using an ABI 7300 real‐time PCR machine. The array data for relative fold changes were analyzed using the SABiosciences PCR Array Data Analysis Software. A separate set of hearts from postexercised mice (24h postexercise; *N* = 6) was used to look at adhesion molecule and extracellular matrix transcripts via the mouse RT^2^ Profiler Extracellular Matrix and Adhesion Molecule PCR array (PAMM‐013ZA, Qiagen).

Protein amounts from all samples were quantified using the BCA assay (ThermoScientific). Fifty micrograms of protein were separated by SDS‐PAGE and transferred onto PVDF membrane via Western blot for quantification of Cleaved PARP: anti‐PARP antibody (9542 and 9546; Cell Signaling, Boston, MA). Chemiluminescent signals were visualized in a FluorChem E imager and final band intensity was quantified using AlphaView software (ProteinSimple, San Jose, CA).

### In vitro recapitulation of exercise cell damage

Secondary to the greater blood volume in rats versus mice, we harvested plasma from exercise‐naïve rats for our in vitro experiments. Neonatal rat cardiomyocytes (NRCMs) were isolated according to standard procedures and cultured on gelatin‐coated plates (Boudou et al. 2012). This isolated cell model provides an important means to analyze the primary effects of exercise on the heart as it avoids differential loading. Cells were plated on 96‐well plates at a density of 1 × 10^4^ cells/well and cultured in DMEM supplemented with 10% FCS, 10 mmol/L Hepes, 2 mmol/L glutamine, 100 U/mL penicillin, and 100 *μ*g/mL streptomycin. In vitro recapitulation of exercise cell damage was performed by culturing NRCMs under 2% sedentary or acute exercise plasma conditions.

Exercise plasma was obtained from eight female (Sprague–Dawley) rats immediately after running on a motorized rodent treadmill at 18 m/min, 0% grade, for 60 min. This exercise protocol is similar in intensity to the mouse exercise protocol as it maintains rats at 60–70% VO_2_ peak, and has been previously used by our group and others (Bedford et al. 1979; Libonati et al. 1997; Roque et al. 2013). Sedentary animal plasma was also collected. Plasma was not pooled for in vitro experiments. Immediately following exercise, animals were anesthetized, and blood was collected in EDTA tubes from the inferior vena cava following a thoracotomy. Isolated plasma was stored in aliquots at −80°C until use.

Cell survival is reported as a measure of viability over cytotoxicity, as assayed with the MultiTox‐Fluor Multiplex Cytotoxicity Assay (G 9200, Promega, Madison, WI). This assay simultaneously measures two protease activities as a measure of cell viability and cytotoxicity, respectively. Briefly, a cell‐permeant substrate is cleaved by live cell protease activity to produce a fluorescent signal proportional to the number of living cells. Similarly, a cell‐impermeant peptide substrate is used to measure dead‐cell protease activity, which is released from cells with compromised membrane integrity.

### Plasma catecholamine assays

Epinephrine and norepinephrine levels in collected plasma were assayed in duplicate using the Bi‐Cat ELISA kit (BCT 31KO2, Eagle Biosciences, Nashua, NH) as per the manufacturer's instructions. Briefly, 300 *μ*L of plasma was used for extraction and acylation of epinephrine and norepinephrine to N‐acylepinephrine and N‐acylnorepinephrine. N‐epinephrine and N‐norepinephrine were assayed by a competitive sandwich ELISA using specific antibodies to each compound.

### *β*‐Adrenergic blockade and antioxidant treatment in NRCMs

The *β*‐adrenergic pathway was explored as a potential mechanism through which exercise lowers cell survival (Viability/Cytotoxicity). To study the *β*‐adrenergic pathway, propranolol (P0884, Sigma Aldrich) was used as a nonselective *β* antagonist and isoproterenol (I6379, Sigma Aldrich) was used as a *β* agonist. NRCMs were treated with both drugs at 1 *μ*mol/L concentration for 20 h. To study how reactive oxygen species may affect cell survival, antioxidants were applied to NRCMs under sedentary or exercise plasma conditions. The antioxidant reagents were purchased from Sigma Aldrich and included D‐ɑ‐Tocopherol succinate (T3126), Tempol (176141), and a cell culture media Antioxidant Supplement (A1345). These were used at 100 *μ*mol/L, 150 *μ*mol/L, and 5× concentrations, respectively. These concentrations were chosen after pilot experiments to establish a suitable drug concentration.

### MSCs in exercise conditions

Rat mesenchymal stem cells (CD29^+^, CD44^+^, CD90^+^, CD11b^−^, CD34^−^, and CD45^−^; Gibco) were cultured under adherent cell culture conditions. MSCs were seeded at 2 × 10^5^ per well in a 96‐well plate and treated with either 2% sedentary or exercise animal plasma in cell culture media for 24 h. Interleukin‐6 (IL‐6), tumor necrosis factor‐alpha (TNF‐*α*), interleukin‐1*β* (IL‐1*β*), and interferon‐gamma (IFN‐*γ*) levels in conditioned media from MSCs treated with 2% sedentary or exercise plasma were quantified with ELISA. For coculture experiments, NRCM were seeded in 24‐well plates and MSCs were seeded in 0.4 *μ*mol/L cell culture inserts (EMD Millipore, Billerica, MA). After 24 h of coculture, NRCM survival was assayed as described above. The BrdU Cell Proliferation Assay Kit (Cell Signaling, #6813) was used per the manufacturer's protocol to determine cell proliferation. A radius migration assay (CBA‐125, Cell Biolabs, San Diego, CA) was used to assess migratory potential of NRCMs in exercise conditions. Chemotaxis of NRCM in response to 2% exercise and sedentary plasma in culture media was measured via Boyden chamber assays.

### Data analysis

A one‐way ANOVA with *t*‐test post hoc analysis was used to compare the temporal changes in cleaved PARP in mouse hearts during the postexercise interval. Unpaired *t*‐tests were used to compare the responses of NRCMs and MSCs to sedentary and exercise plasma conditions individually and in coculture conditions. The probe sets from gene arrays were ranked according to their most significant *P*‐values, which were then adjusted by using the Benjamini and Hochberg (BH) method. Significance value was set at an alpha level of *P* ≤ 0.05. Data are reported as the mean ± SEM. All analyses were performed using SPSS (Chicago, IL).

## Results

### Acute exercise and temporal apoptosis in mice

**Figure 1:**
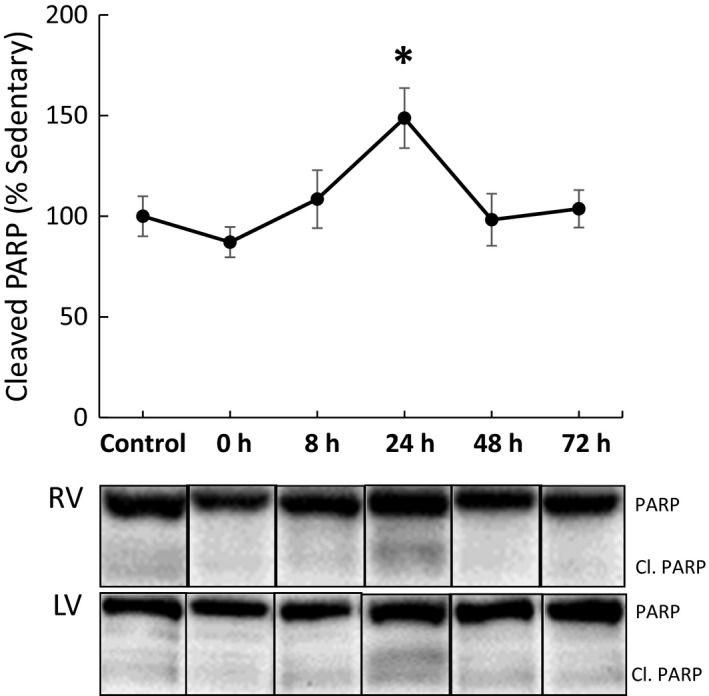
Whole heart apoptosis 24 h after exercise. Temporal apoptosis, as measured through cleaved PARP, was significantly increased 24 h postexercise and recovered to sedentary control levels following this time point. RV, right ventricle; LV, left ventricle; Control, sedentary control; 0, exercise animals sacrificed immediately after exercise; 8, exercise animals sacrificed 8 h postexercise; 24, exercise animals sacrificed 24 h postexercise; 48, exercise animals sacrificed 48 h postexercise; 72, exercise animals sacrificed 72 h postexercise (*P ≤ 0.05 from sedentary control).

Protein levels of cleaved PARP (poly ADP‐ribose polymerase), a marker of apoptosis (Soldani et al. 2001), were significantly increased in the myocardium 24 h postexercise relative to the sedentary controls and returned to sedentary levels by 48 and 72 h postexercise (Fig. 1). Of the 84 genes analyzed in the RT^2^ Profiler PCR array, a total of 11 relevant genes displayed alterations of twofold up‐ or downregulation with the exercise treatment (Table 1). An upregulation pattern with a fold value >2 occurred in genes Bcl2l2, Cidea, Fasl, Tnfrsf11b/OPG, and Trp73 at 72 h postexercise (Table 1), whereas genes Trp73, Bcl2a1a, Bid, Cd40lg, Nme5, Tnf, and Naip1 were downregulated with fold‐change values less than −2 at 8 h postexercise. Fold changes for all assayed genes are shown in Table S1.

**Table 1:** Summary apoptosis array in mouse myocardium

Symbol	Description	Fold change (compared to control)				
		0HR	8HR	24HR	48HR	72HR
Bcl2a1a	B‐cell leukemia/lymphoma 2‐related protein A1a		−2.0431			
Bcl2 l2	Bcl2‐like 2					2.4361
Bid	BH3 interacting domain death agonist	−2.2903				
Cd40lg	CD40 ligand	−3.4419	−2.1382	−2.3453	−4.1212	−3.0682
Cidea	Cell death‐inducing DNA fragmentation factor, alpha subunit‐like effector A					2.7106
Fasl	Fas ligand (TNF superfamily, member 6)					3.0694
Naip1	NLR family, apoptosis inhibitory protein 1				−2.0706	
Nme5	Nonmetastatic cells 5, protein expressed in nucleoside‐diphosphate kinase		−5.2929			
Tnf	Tumor necrosis factor	−2.5708	−2.9582			
Tnfrsf11b	Tumor necrosis factor receptor superfamily, member 11b (osteoprotegerin)		2.3673	2.9286		6.7222*
Trp73	Transformation‐related protein 73		−2.1761			3.5429

*P ≤ 0.05 from sedentary control.

A separate study by our group demonstrated that exercise can increase retention of bone marrow mononucleated cells in the myocardium (Chirico et al. 2015). Thus, we also performed a PCR array for extracellular matrix and cell adhesion molecules. Vascular cell adhesion molecule 1 (Vcam‐1), intercellular adhesion molecule 1 (Icam‐1), p‐selectin (Selp), and Mmp‐12, ‐1a, and ‐2 transcripts were all upregulated twofold or higher at the 24‐h time point, whereas Integrins (Itga2, Itgax, Itgb2) and thrombospondin1 (Thbs1) were downregulated twofold or lower (Table 2). Fold changes for all assayed genes are shown in Table S2.

**Table 2:** Summary matrix remodeling in mouse myocardium 24 h postexercise

Gene	Description	Fold change (compared to control)
Cntn1	Contactin 1	2.28835
Col3a1	Collagen, type III, alpha 1	−2.2674
Col4a3	Collagen, type IV, alpha 3	−2.8116
Icam1	Intercellular adhesion molecule 1	2.4127
Itga2	Integrin alpha 2	−4.7288
Itgax	Integrin alpha X	−5.4835
Itgb2	Integrin beta 2	−2.0069
Itgb3	Integrin beta 3	2.1812
Mmp13	Matrix metallopeptidase 13	−2.0127
Mmp1a	Matrix metallopeptidase 1a (interstitial collagenase)	2.3986
Mmp2	Matrix metallopeptidase 2	2.95695
Mmp3	Matrix metallopeptidase 3	−4.6926
Ncam2	Neural cell adhesion molecule 2	5.51
Selp	Selectin, platelet	3.3025
Spock1	Sparc/osteonectin, cwcv, and kazal‐like domains proteoglycan 1	−2.1047
Spp1	Secreted phosphoprotein 1	−3.2229
Syt1	Synaptotagmin I	−2.6087
Thbs1	Thrombospondin 1	−3.8149
Vcam1	Vascular cell adhesion molecule 1	3.99435

### Neonatal rat cardiomyocyte culture and exercise plasma

**Figure 2:**
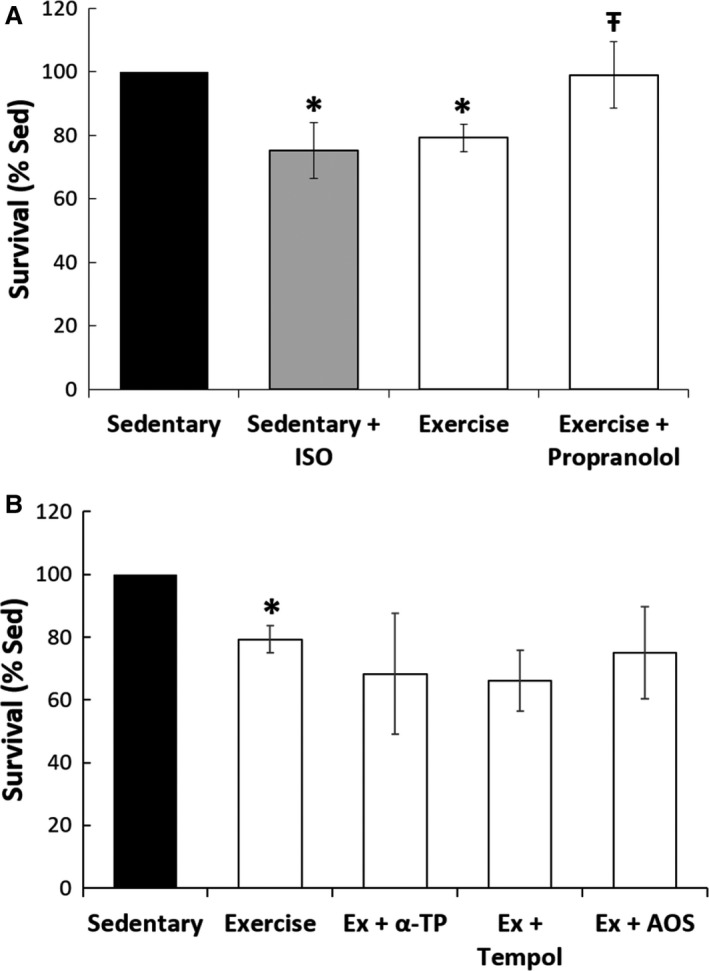
Neonatal rat cardiomyocyte culture with catecholamine antagonism and antioxidants. (A) Beta‐blockade. Addition of isoproterenol (ISO) to the sedentary plasma significantly reduced NRCM survival to levels comparable to the exercise condition. Addition of propranolol to NRCMs with exercise plasma improved the survival of NRCMs to sedentary levels (*P ≤ 0.05 from sedentary control, Ŧ *P* ≤ 0.05 from exercise). (B) Antioxidant treatment. Addition of mechanistically different antioxidants (α‐TP, alpha‐tocopherol; AOS, antioxidant supplement) provided no protection against acute exercise damage.

We investigated humoral and oxidative‐mediated cell death in vitro using isolated NRCMs cultured with plasma from sedentary and acutely exercised rats over a 24 h interval, a time point selected in order to simulate the in vivo apoptosis response. Plasma concentrations of epinephrine and norepinephrine tended to be increased following exercise (data not shown). NRCMs cultured with 2% plasma from exercise animals showed a decreased survival of 79% relative to sedentary treatment (*P* ≤ 0.05, Fig. 2A). *β*‐adrenergic stimulation with isoproterenol in sedentary plasma conditions significantly decreased survival of NRCMs (*P* ≤ 0.05) to levels comparable to those of the exercise condition (75% vs. 79%), and *β*‐adrenergic blockade of exercise conditions with propranolol rescued survival levels to near sedentary conditions (99% vs. 100%, Fig. 2A). A variety of antioxidants, including ɑ‐Tocopherol (a vitamin E derivative), Tempol (a membrane‐permeable radical scavenger), catalase, and N‐2‐mercaptopropionyl glycine (MPG, free radical scavenger), and a media antioxidant supplement (AOS), were added to exercise plasma media to investigate concurrent pathways of cell death due to acute exercise. None of these treatments improved NRCMs survival under exercise plasma conditions (Fig. 2B).

### Acute exercise and stem cells

**Figure 3:**
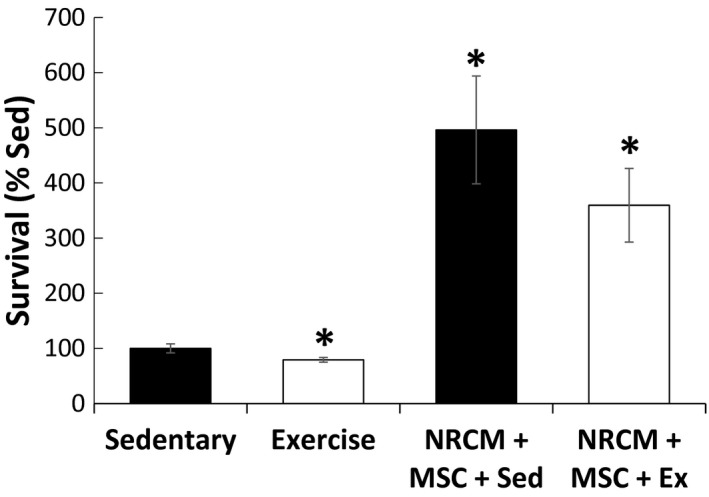
Co‐culture and migration of MSCs with NRCM. Co‐culture of MSCs with NRCMs significantly increased NRCM survival in sedentary conditions and exercise conditions. (*P ≤ 0.05 from sedentary control).

**Figure 4:**
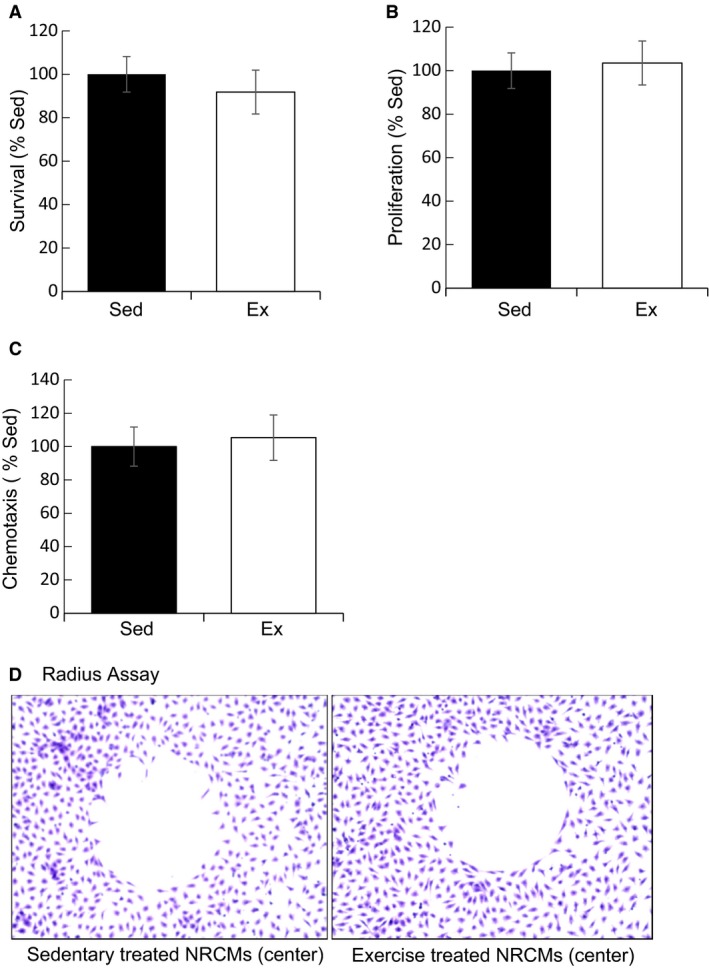
Effects of exercise conditions on MSC. (A) Survival. MSC survival was similar in 2% sedentary and exercise conditions. (B) Proliferation. MSC proliferation as measured via BrdU incorporation showed no differences between sedentary and exercise conditions. (C) Chemotaxis. Boyden chamber migration of MSCs alone showed no difference between experimental conditions. (D) Radius Assay. MSCs exhibited no differential migration towards sedentary or exercise treated NRCM lysates (center).

**Figure 5:**
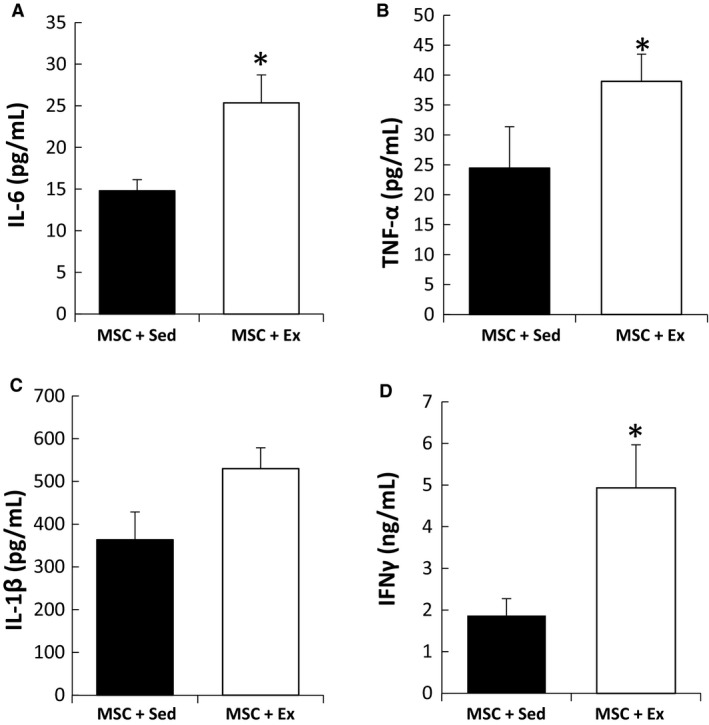
Inflammatory response of MSCs to exercise conditions. ELISA assay revealed that exercise elicited an inflammatory response from MSCs as determined by higher levels of (A) IL‐6 (25.4 vs 14.8 pg/mL, *P* ≤ 0.01), (B) TNF‐α (40.0 vs 24.5 pg/ml, *P* ≤ 0.05), (C) IL1‐b (529 vs 363 pg/mL, P = 0.16) and (D) IFN‐γ (4.93 vs 1.9 ng/ml, *P* ≤ 0.01) relative to sedentary. (*P ≤ 0.05 from control).

As MSCs are involved in tissue repair, the effect of exercise on MSCs was studied in vitro. Coculture experiments of NRCMs with MSCs showed that MSCs can increase NRCM survival by up to five times, and that this therapeutic effect was independent of sedentary or exercise conditions (Fig. 3). Moreover, MSC survival, proliferation, and chemotaxis showed no significant differences between sedentary and exercise plasma conditions (Fig. 4A–C), and no differential migration toward sedentary or exercise‐treated NRCM lysate was observed (Fig. 4D). An ELISA assay revealed that exercise elicited an inflammatory response from MSCs as indicated by higher levels of IL‐6 (25.4 vs. 14.8 pg/mL, *P* ≤ 0.01), TNF‐*α* (40.0 vs. 24.5 pg/mL, *P* ≤ 0.05), IL1‐*β* (529 vs. 363 pg/mL, *P* = 0.16), and IFN‐*γ* (4.93 vs. 1.9 ng/mL, *P* ≤ 0.01) relative to sedentary (Fig. 5A–D).

## Discussion

For this study, we hypothesized that a single bout of treadmill exercise would induce apoptosis and prompt repair mechanisms by MSCs on the heart. Our results in rodents showed that myocardial apoptosis was induced in the heart 24 h postexercise, and was in part due to catecholaminergic stress, but not oxidative stress. Even though exercise conditions prompted an inflammatory response in MSCs, neither their survival, proliferation, chemotaxis, nor their protective signaling toward cardiomyocytes was different from sedentary conditions. Together, these results suggest that despite increased cardiomyocyte death with acute exercise, exercise does not further activate MSCs to protect the heart.

In this study, cleaved PARP, a marker of apoptosis, was elevated 24 h after exercise and returned to baseline by 48 h postexercise. PARP is activated by DNA damage as well as posttranslational modifications elicited by various signaling pathways. The PARylation of proteins serves diverse and important functions in the cells, including repair of DNA breaks and the regulation of transcriptional factors (Bürkle and Virág 2013). This pathway is unique in that it is involved both in signaling cell death by apoptosis, as well as enhancing recovery of cells from DNA damage (Bürkle and Virág 2013). Functionally, our group has shown that an exercise paradigm similar to that used in this study resulted in worsened diastolic rigor during ischemia and gradual recovery by 24 h postexercise (Reger et al. 2012). Our findings are consistent with previous studies which have shown that aerobic exercise results in myocardial dysfunction and cardiac injury even in healthy individuals (Shave et al. 2010). For example, studies following recreational runners immediately following the completion of marathons demonstrated a reduction in diastolic function and an increase in plasma cardiac troponin levels (Whyte et al. 2005; Trivax et al. 2010). Mechanisms such as mechanical stress, catecholamine toxicity, oxidative stress, and/or acute inflammatory cytokine activation have all been hypothesized to play a role in the exercise‐induced cardiomyocyte damage (Bishopric et al. 2001; Scharhag et al. 2008).

In this study, we tested the above potential mechanisms of exercise‐induced apoptosis in the heart. We observed increased apoptosis in both the right and left ventricles along with a small number of temporal apoptotic‐related transcript shifts. Sympathetic outflow is well known to increase with aerobic exercise, and in this study we found that plasma concentrations of epinephrine and norepinephrine were increased following exercise. Hence, we tested if we could recapitulate the increased cardiomyocyte cell death observed in whole hearts using a model of quiescent neonatal rat cardiomyocytes exposed to exercise plasma in cell culture conditions. We found that neonatal rat cardiomyocyte survival was decreased by plasma from exercised animals compared to those in sedentary conditions, suggesting a role of plasma‐borne factors in the whole heart response. We then investigated whether treatment with antioxidants or nonspecific *β*‐adrenergic blockade could mitigate exercise apoptosis at the 24‐h time point. While our results showed that antioxidant treatment did not improve cardiomyocyte survival, we found that *β*‐adrenergic blockade with propranolol improved survival levels toward sedentary levels. This suggests that catecholaminergic stress is, in part, responsible for the pattern of postexercise myocardial apoptosis. These results corroborate and define the transient cardiomyocyte injury observed by Wallner et al. (2016).

To understand transcriptional changes in apoptotic pathways as a result of strenuous acute exercise, hearts from exercised animals were harvested. We focused only on those transcripts that were either twofold up‐ or downregulated. In our study, the tumor necrosis factor receptor superfamily member 11b (Tnfrsf11b) was upregulated at 8, 24, and 72 h postexercise. Ligands of the tumor necrosis factor superfamily (TNFSF) are regulatory in apoptosis, and may be involved in the regulation of apoptosis in heart failure patients (Ueland et al. 2012). TNFRSF11b, also known as oseoprotegerin (OPG), can potentially interact with TNF‐related apoptosis‐inducing ligand (TRAIL), protecting cells from apoptosis (Ueland et al. 2012). TNFRSF11b may also inhibit the activity of receptor activator of NF‐KB ligand (RANKL), which is responsible for prolonging dendritic cell survival and increasing the release of inflammatory cytokines (Ueland et al. 2012). The CD40 ligand (Cd40lg), also a member of the TNF superfamily, was downregulated at all time points after exercise in our study. Its signaling role in cardiac apoptosis is largely unknown, but increased levels have been implicated in heart failure and inflammatory conditions, as its increased expression aids immune cell binding via CD40 (Ueland et al. 2005; Elgueta et al. 2009). While our studies did not tease out the role of these transcriptional changes in the apoptotic response, the temporal nature of these genes after exercise may have influenced the postexercise apoptosis seen in each ventricle.

After we established that acute exercise induced myocardial apoptosis in vivo and in vitro, we went on to study whether exercise conditions could stimulate the therapeutic potency of MSCs. In vitro treatment (24 h) of MSCs with 2% plasma from sedentary or exercise animals revealed no significant changes in survival, proliferation, or chemotaxis. However, treatment with 2% exercise plasma increased cytokine secretions from MSCs, namely, IL‐6, TNF‐*α*, IL‐1*β*, and IFN‐*γ*. Our coculture experiments revealed that MSCs increased the survival of cardiomyocytes by up to 500% despite the exercise‐induced proinflammatory secretions by MSCs, although our studies did not determine whether MSC‐mediated dilution of catecholamines caused enhanced cell survival in NRCMs. Our results are in accord with a recent study by Lavorato et al. (2016), who showed that MSCs and chronic exercise training may individually provide benefit to the infarcted heart, but are not summative in their effects. It remains unclear whether acute exercise could provide an adjuvant benefit to MSC therapy.

IL‐6 is known to increase anti‐inflammatory cytokine production such as IL‐1ra (interleukin‐1 receptor agonist) and IL‐10 (interleukin‐10), and inhibit further proinflammatory TNF‐*α* production postexercise (Petersen and Pedersen 2005). Conversely, TNF‐*α*, IL‐1*β*, and IFN‐*γ* are proinflammatory cytokines involved in the acute inflammatory phase of tissue damage and can stimulate or modulate the release of additional proinflammatory signaling molecules and recruit macrophages (Sugarman et al. 1985; Krampera et al. 2006; Ryan et al. 2007; DelaRosa et al. 2009). Interestingly, the concomitant presence of IFN‐*γ* and TNF‐*α* has been found to induce ICAM‐1 and VCAM‐1 (Ren et al. 2010), transcripts which showed increased expression in our study. Furthermore, many of the upregulated genes we found have protein products with known interactions with MSCs. For example, VCAM1 and ICAM1 are involved in MSC immunosuppression via cell–cell contact (Ren et al. 2010; Yagi et al. 2010), and MMP‐13, ‐1a, and ‐2 are bound and activated by MSCs during the differentiation pathway (Lozito et al. 2014). MMPs have a critical role in defining the differentiation of MSCs, and tissue‐specific overexpression may define transdifferentiation of MSCs into a specific cell lineage (Almalki and Agrawal 2016).

We also saw upregulation of p‐selectin (Selp) transcripts, which mediate interactions between blood cells and the endothelium (Blann et al. 2003), and a downregulation of thrombospondin 1 (Thbs1), a transcript involved in antiangiogenic activity, and integrins (Itga2, Itgax, Itgab2), signal transducers involved in determining endothelial cell morphology, migration, proliferation, differentiation, and survival that are known to be reduced during heart failure and hypertension (Ross and Borg 2001; Manso et al. 2009). Integrin transcripts (Itga2, Itgax, Itgbs) and thrombospondin 1 (Thsp1) were downregulated, suggesting modulation of angiogenic pathways.

While our observations help in understanding the role of acute exercise on the heart, there are several limitations to our work. First, we acknowledge our limited sample sizes in the apoptosis response to exercise, and future in vivo experiments will be necessary to determine if administration of *β*‐adrenergic pathway antagonists abrogate cardiomyocyte injury with exercise and whether mechanisms of injury beyond those seen in our in vitro model may be involved. Second, although the paracrine‐mediated protective effect of MSCs on cardiomyocytes was not significantly different between sedentary and exercise conditions in vitro, our experiments did not study potential therapeutic capacity as a result of direct cell–cell contact or identify whether the transcriptional changes in matrix remodeling proteins described above may cause increased MSC recruitment or retention to improve therapeutic potency in the whole heart. Furthermore, it would be interesting to characterize the activity of stem cell populations beyond MSCs, such as cardiac progenitor cells, in the exercised myocardium.

Despite these limitations, our findings hold translational significance to humans in understanding the stress–response characteristics of the heart to aerobic exercise and further our understanding as to whether acute exercise might be useful in potentiating the effects of MSCs in the myocardium following intensive exercise. Our study also suggests that *β*‐adrenergic signaling may be one of the main endocrine factors resulting in cardiac damage in the postexercise window.

## Conflict of Interest

No conflicts of interest, financial or otherwise, are declared by the author(s).

## Data Accessibility
